# Supplementary material for: Proteo-genomic characterization of virus-associated liver cancers reveals potential subtypes and therapeutic targets
Source: Nat Commun. 2022 Oct 29;13:6481. doi: 10.1038/s41467-022-34249-x (PMC9617926; doi:10.1038/s41467-022-34249-x)
Supplement: Supplementary file 1 — Supplementary Information [file 41467_2022_34249_MOESM1_ESM.pdf]

Supplementary Information

Proteo-genomic Characterization of Virus-associated Liver Cancers Reveals Potential Subtypes and Therapeutic Targets

Masashi Fujita[1], Mei-Ju May Chen[2], Doris Rieko Siwak[3], Shota Sasagawa[1], Ayako Oosawa-Tatsuguchi[1], Koji Arihiro[4], Atsushi Ono[5], Ryoichi Miura[5], Kazuhiro Maejima[1], Hiroshi Aikata[5], Masaki Ueno[6], Shinya Hayami[6], Hiroki Yamaue[6], Kazuaki Chayama[5], Ju-Seog Lee[3], Yiling Lu[3], Gordon B. Mills[7], Han Liang[2], Satoshi S. Nishizuka[8], and Hidewaki Nakagawa[1*]

Supplementary Figure 1. Evaluation of the cluster numbers using the ConsensusClusterPlus package.

Supplementary Figure 2. Similarity of mRNA-protein correlations between cancer types.

Supplementary Figure 3. Characteristic proteins of each proteomic subclass.

Supplementary Figure 4. Validation in the TCGA-LIHC cohort.

Supplementary Figure 5. Immunohistochemistry of CD45, CD4, and CD8 in the RIKEN dataset.

Supplementary Figure 6. Protein expression levels of HIF1A.

Supplementary Figure 7. mTOR activity and its inhibition in liver cancer cell lines.

Supplementary Figure 8. Expression levels of poor-prognostic proteins.

Supplementary Figure 9. Expression levels of good-prognostic proteins.

Supplementary Figure 10. Gene set enrichment analysis between HCV-positive and HCV-negative tumors.

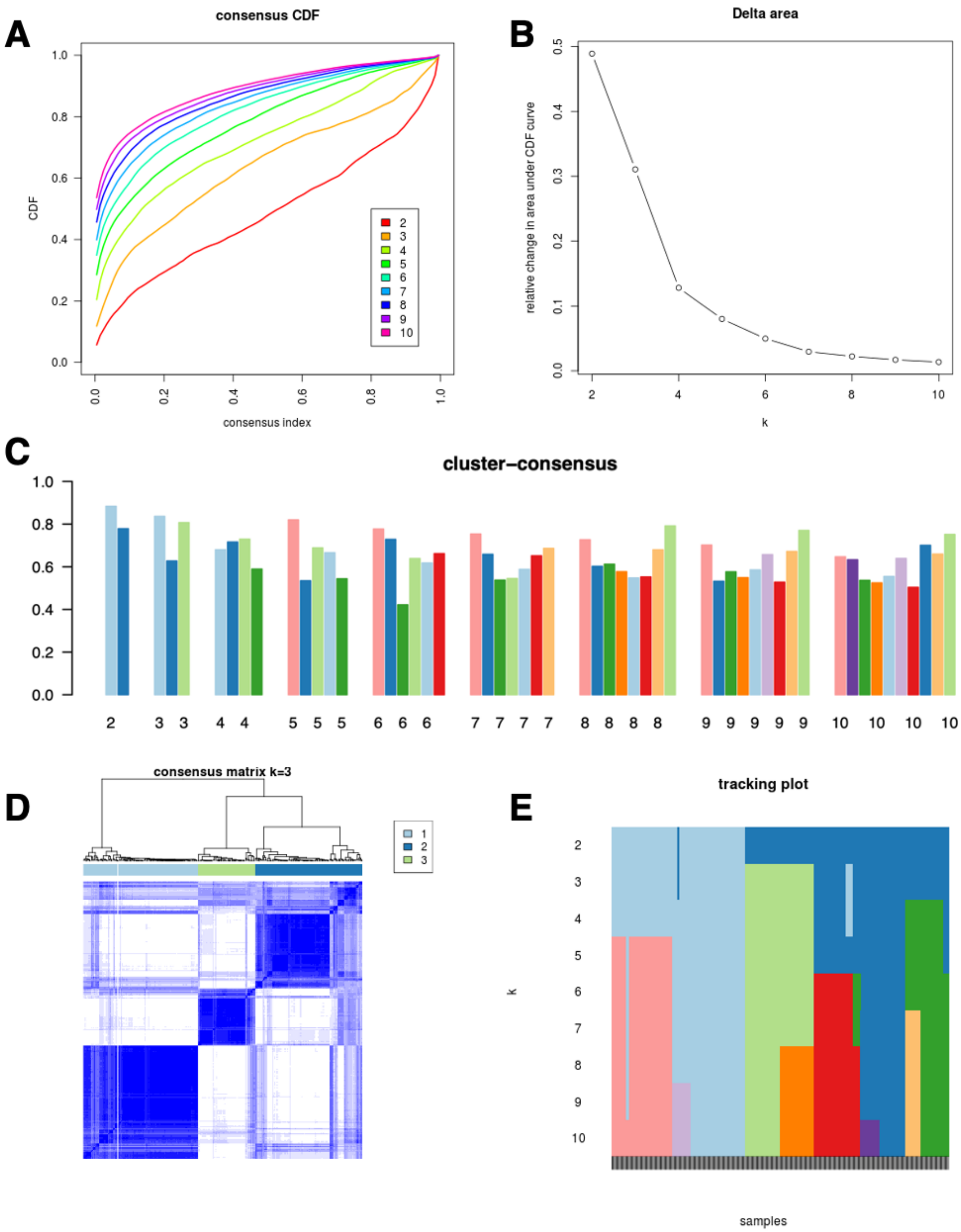

Supplementary Figure 1. Evaluation of the cluster numbers using the ConsensusClusterPlus package. (**A**) Consensus cumulative distribution function (CDF) for each cluster number *k*. (**B**) Relative change in area under the CDF curve comparing *k* and *k* − 1. (**C**) Cluster-consensus value of clusters for *k* = 2, 3, …, 10. (**D**) Heatmap of the consensus matrices for *k* = 3. (**E**) Cluster assignment of tumor samples tracked with increasing *k*. The columns show 259 tumor samples, and their cluster membership are color coded.

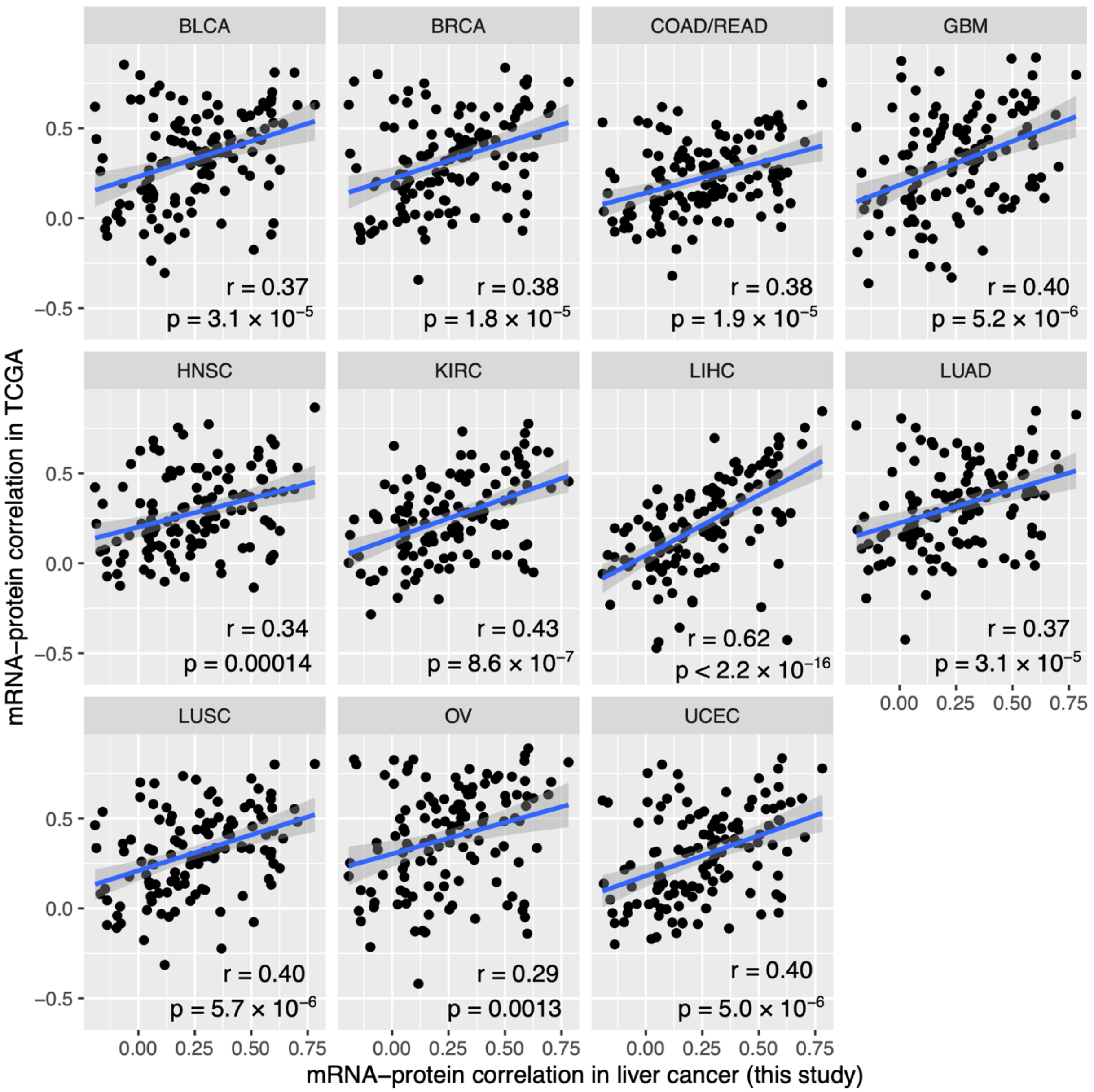

Supplementary Figure 2. Similarity of mRNA-protein correlations between cancer types. Each dot represents a matched pair of mRNA and protein. The x axis shows Spearman's correlation coefficient between the mRNA and protein abundance in this study. The y axis shows Spearman's correlation coefficient between the mRNA and protein abundance in TCGA cohorts. *r*, Spearman's correlation coefficient between the mRNA-protein correlations in this study and those in TCGA. Blue lines show linear regressions. Error bands show 95% confidence intervals. The p-values were computed by two-sided Spearman's correlation test without adjustments for multiple comparisons. Source data are provided as a Source Data file.

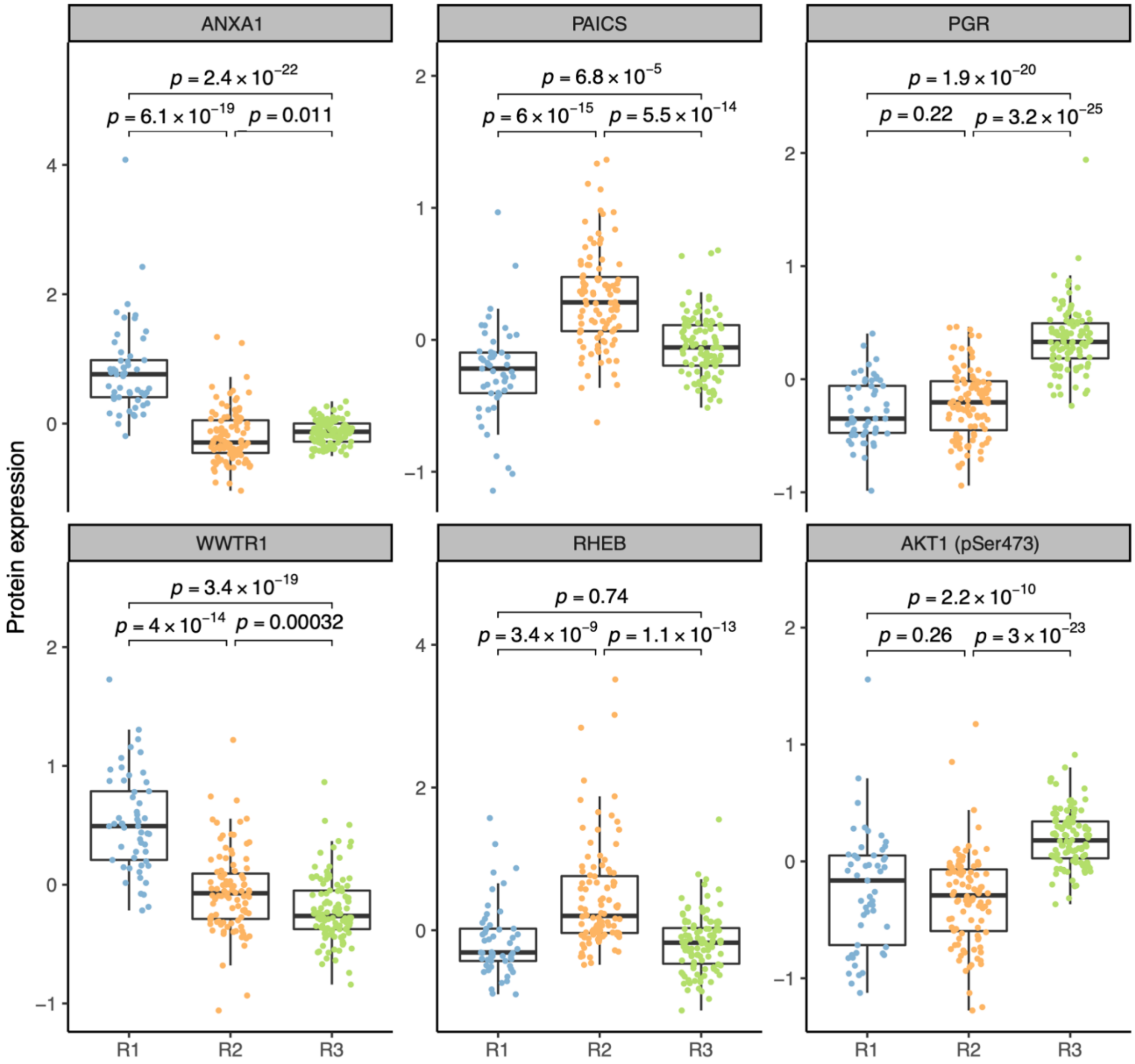

Supplementary Figure 3. Characteristic proteins of each proteomic subclass. ***, $p < 0.001$; **, $p < 0.01$; *, $p < 0.05$; NS, $p \geq 0.05$. The p-values were computed using two-sided Wilcoxon rank sum test. Sample sizes are n = 53 in R1, n = 100 in R2, and n = 106 in R3. Center of box shows the median. Lower and upper bounds of box are the first and third quartiles, respectively. Minima and maxima are the farthest values within 1.5 times the inter-quartile range from the bounds of box. Whiskers are drawn from the bounds of box to the extrema. Source data are provided as a Source Data file.

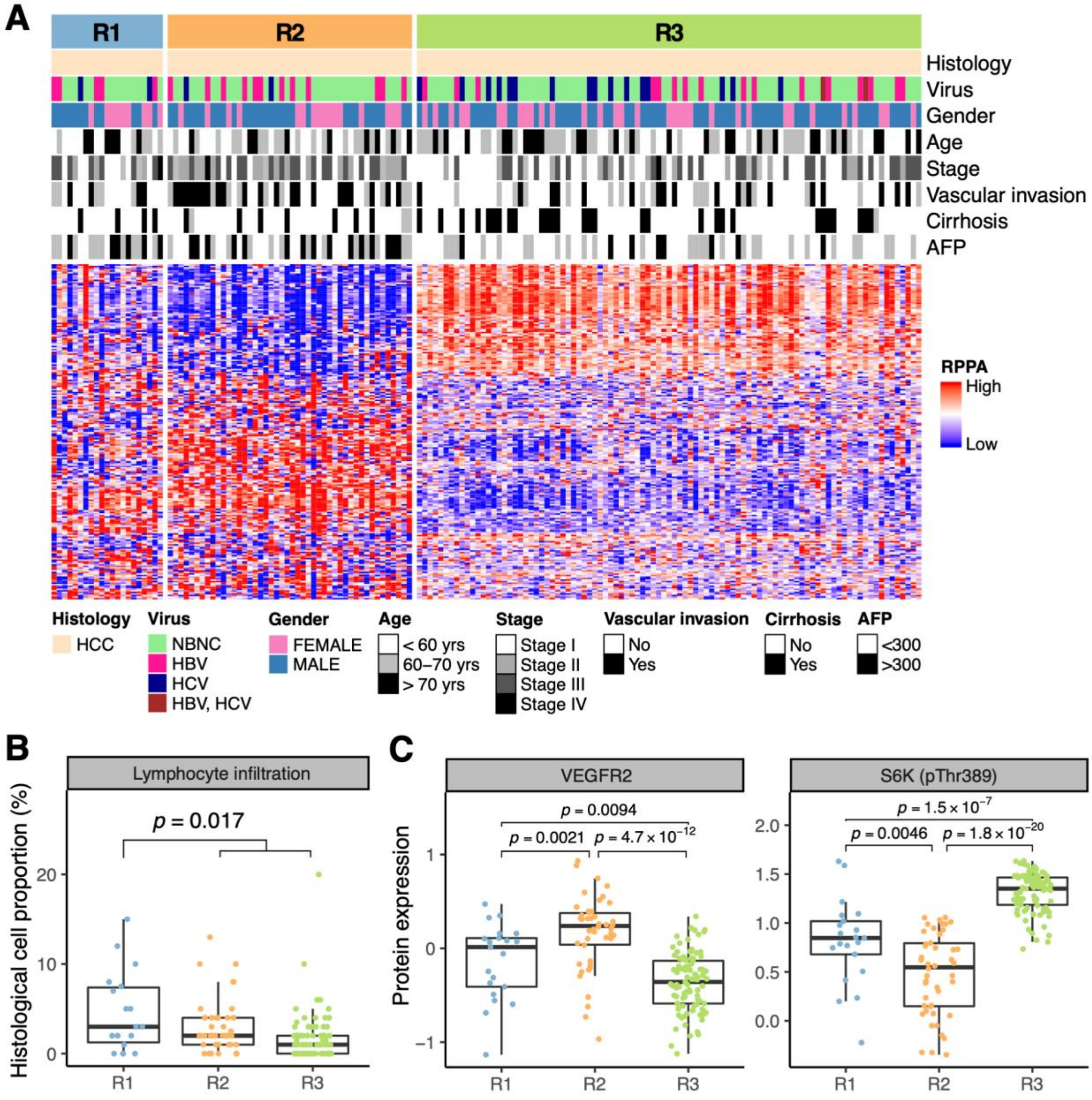

Supplementary Figure 4. Validation in the TCGA-LIHC cohort. (**A**) Proteomic subclasses R1–R3, clinical features, and expression levels of 218 proteins in 162 tumors of TCGA-LIHC. HCC, hepatocellular carcinoma; NBNC; non-hepatitis B non-hepatitis C. (**B**) Abundance of tumor-infiltrating lymphocytes as histologically measured with hematoxylin & eosin stained slides. Histological measurements were available for n = 18, n = 43, and n = 91 biologically independent samples in R1, R2, and R3, respectively. (**C**) Protein expression levels measured by RPPA. ***, $p < 0.001$; **, $p < 0.01$; *, $p < 0.05$; NS, $p \geq 0.05$. The p-values were computed using two-sided Wilcoxon rank sum test. Sample sizes are 21 in R1, 46 in R2, and 95 in R3. Center of box shows the median. Lower and upper bounds of box are the first and third quartiles, respectively. Minima and maxima are the farthest values within 1.5 times the inter-quartile range from the bounds of box. Whiskers are drawn from the bounds of box to the extrema. Source data are provided as a Source Data file.

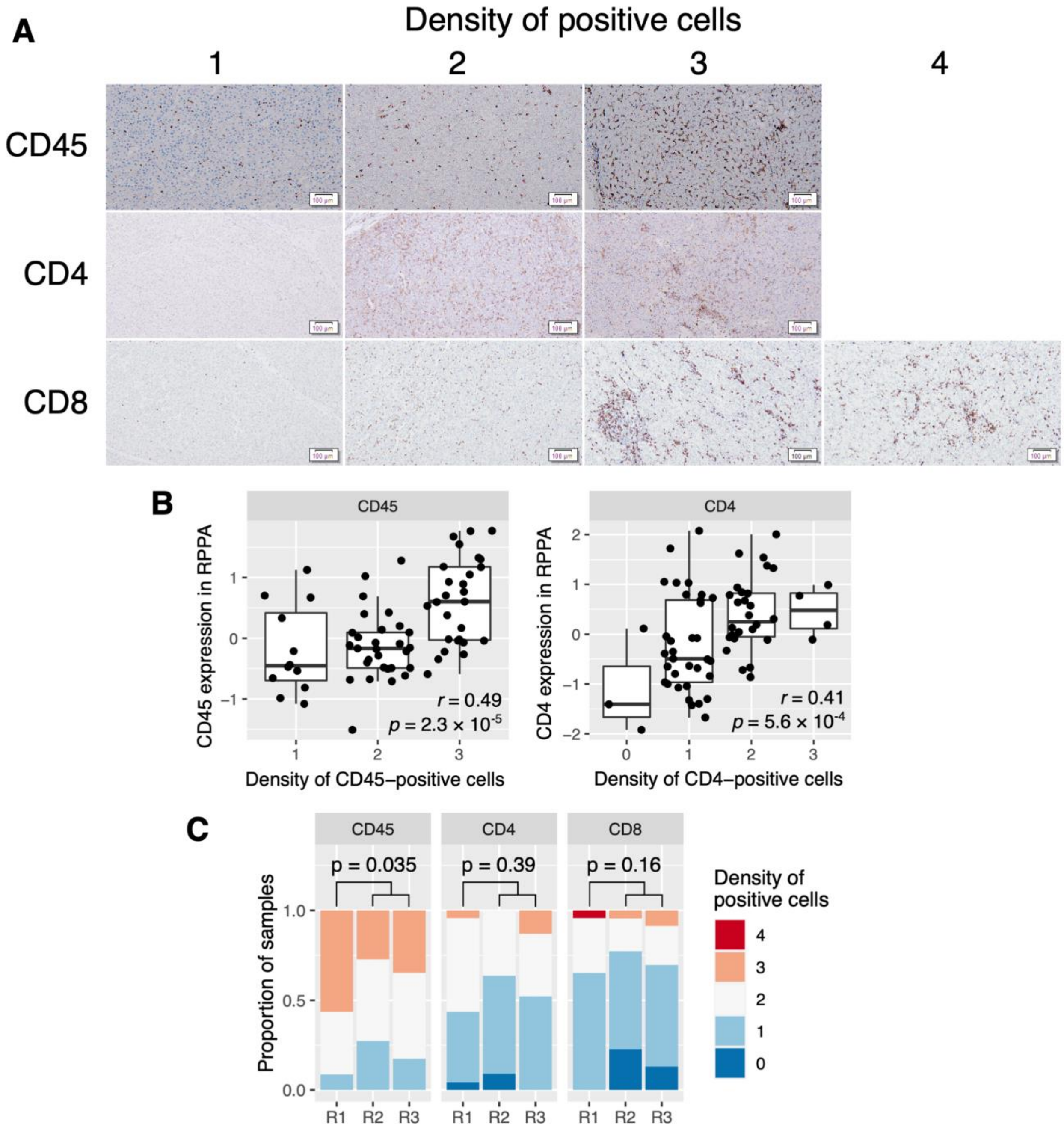

Supplementary Figure 5. Immunohistochemistry of CD45, CD4, and CD8 in the RIKEN dataset. Among tumors analyzed with RPPA, 23 tumors of R1, 22 tumors of R2, and 23 tumors of R3 were randomly selected, and immunohistochemistry was performed. Density of positively stained cells was evaluated in a semi-quantitative manner. (**A**) Representative images of positive cell density for CD45, CD4, and CD8 staining. For each antibody and each tumor, one slide was prepared. All tissue area in the slide was inspected for cell density evaluation. Scale bars show 100 μm. (**B**) Positive cell density and protein expression measured by RPPA. Each dot represents a tumor sample. Sample size was n = 68. *r*, Spearman's correlation coefficient. The p-values were computed by two-sided Spearman's correlation test. CD8 was not included in the antibody set of RPPA. Center of box shows the median. Lower and upper bounds of box are the first and third

quartiles, respectively. Minima and maxima are the farthest values within 1.5 times the inter-quartile range from the bounds of box. Whiskers are drawn from the bounds of box to the extrema. (**C**) Proteomic subclasses and positive cell density. The y axis shows proportion of samples in each level of positive cell density. *, $p < 0.05$; NS, $p \geq 0.05$. The p-values were computed using two-sided Wilcoxon rank sum test. Source data are provided as a Source Data file.

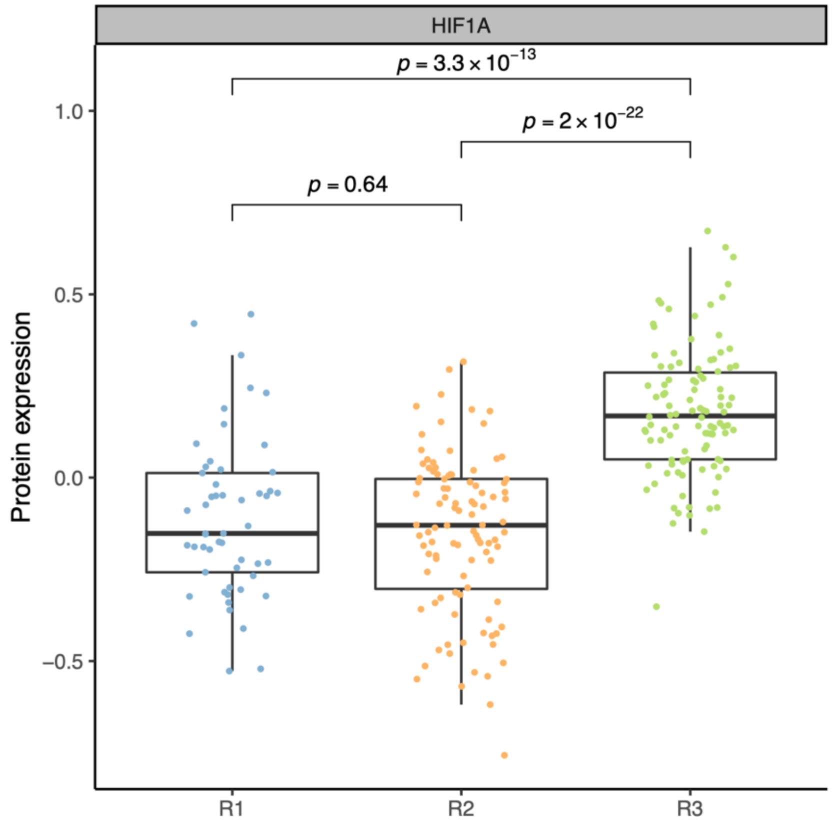

Supplementary Figure 6. Protein expression levels of HIF1A. ***, $p < 0.001$; **, $p < 0.01$; *, $p < 0.05$; NS, $p \geq 0.05$. The *p*-values were computed using two-sided Wilcoxon rank sum test. Sample sizes are n = 53 in R1, n = 100 in R2, and n = 106 in R3. Center of box shows the median. Lower and upper bounds of box are the first and third quartiles, respectively. Minima and maxima are the farthest values within 1.5 times the inter-quartile range from the bounds of box. Whiskers are drawn from the bounds of box to the extrema. Source data are provided as a Source Data file.

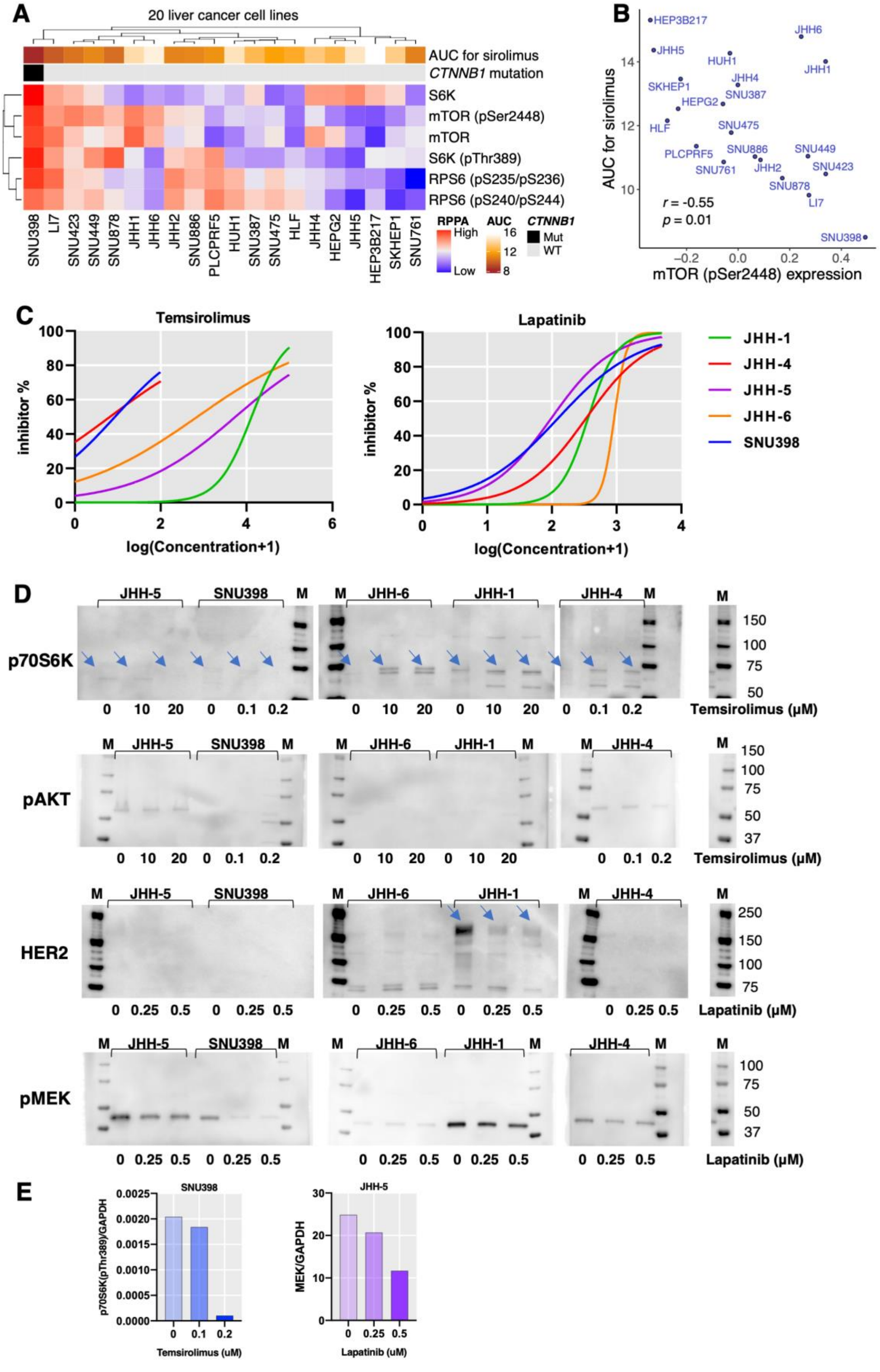
A
20 liver cancer cell lines
AUC for sirolimus
CTNNB1 mutation
S6K
mTOR (pSer2448)
mTOR
S6K (pThr389)
RPS6 (pS235/pS236)
RPS6 (pS240/pS244)
SNU398 LI7 SNU423 SNU449 SNU878 JHH1 JHH6 JHH2 SNU886 PLCPRF5 HUH1 SNU387 SNU475 HLF JHH4 HEPG2 JHH5 HEP3B217 SKHEP1 SNU761
RPPA High Low
AUC 16 12 8
CTNNB1 Mut WT
B
AUC for sirolimus
mTOR (pSer2448) expression
r = -0.55
p = 0.01
HEP3B217 JHH5 HUH1 JHH6 JHH1 SKHEP1 JHH4 SNU387 HEPG2 HLF SNU475 PLCPRF5 SNU886 SNU449 SNU761 JHH2 SNU423 SNU878 LI7 SNU398
C
Temsirolimus
Lapatinib
inhibitor %
log(Concentration+1)
JHH-1
JHH-4
JHH-5
JHH-6
SNU398
D
p70S6K
pAKT
HER2
pMEK
JHH-5 SNU398 JHH-6 JHH-1 JHH-4 M
Temsirolimus (μM)
Lapatinib (μM)
0 10 20 0 0.1 0.2
0 0.25 0.5
E
SNU398
p70S6K(pThr389)/GAPDH
Temsirolimus (uM)
JHH-5
MEK/GAPDH
Lapatinib (uM)

Supplementary Figure 7. mTOR activity and its inhibition in liver cancer cell lines. (**A**) Expression and phosphorylation levels of mTOR pathway proteins in 20 liver cancer cell lines. The data were extracted from RPPA measurement of CCLE. Area under the curve (AUC) for sirolimus, which was computed from dose response curves of growth inhibition assays, was obtained from the CTRP project. The lower AUC, the more potent inhibition of cell growth by the drug. Mutation of *CTNNB1* was found only in the SNU398 cell line. (**B**) Expression levels of mTOR (pSer2448) and AUC for sirolimus in 20 liver cancer cell lines. Spearman's correlation between them is shown. The p-value was computed by two-sided Spearman's correlation test. (**C**) Dose-dependent growth inhibition by temsirolimus and lapatinib on five liver cancer cell lines. (**D**) Five cell lines were treated with various concentrations of temsilorimus or lapatinib for 4 days. The expression levels of the mTOR pathway components p70S6K(Thr389) and pAKT, and HER2 pathway components HER2 and pMEK were examined by western blotting. Independent experiments were performed in triplicate. (**E**) The signal of the western blotting of JHH-5 and SNU398. Source data are provided as a Source Data file.

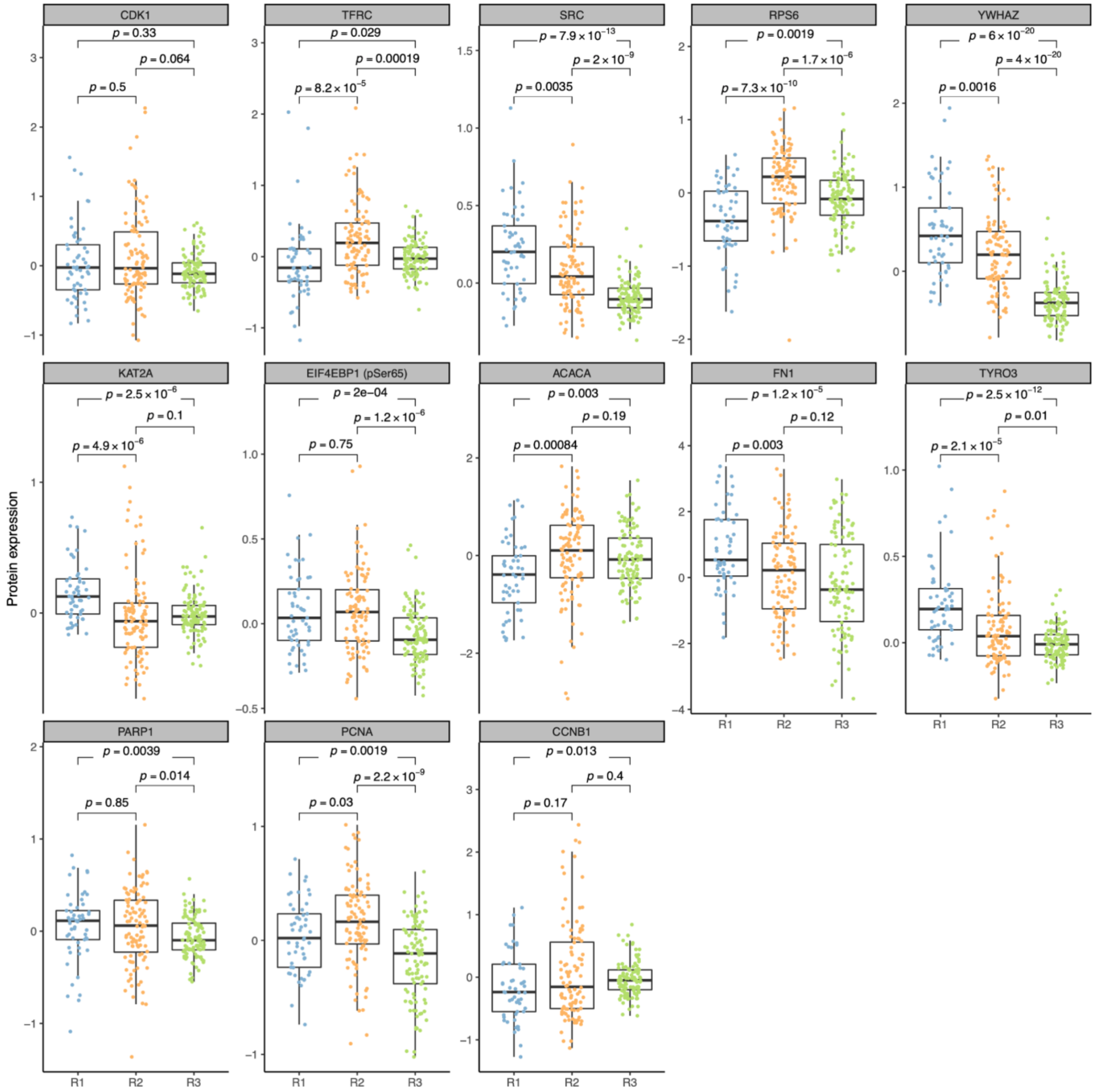

Supplementary Figure 8. Expression levels of poor-prognostic proteins. ***, $p < 0.001$; **, $p < 0.01$; *, $p < 0.05$; NS, $p \geq 0.05$. The p-values were computed using two-sided Wilcoxon rank sum test. Sample sizes are n = 53 in R1, n = 100 in R2, and n = 106 in R3. Center of box shows the median. Lower and upper bounds of box are the first and third quartiles, respectively. Minima and maxima are the farthest values within 1.5 times the inter-quartile range from the bounds of box. Whiskers are drawn from the bounds of box to the extrema. Source data are provided as a Source Data file.

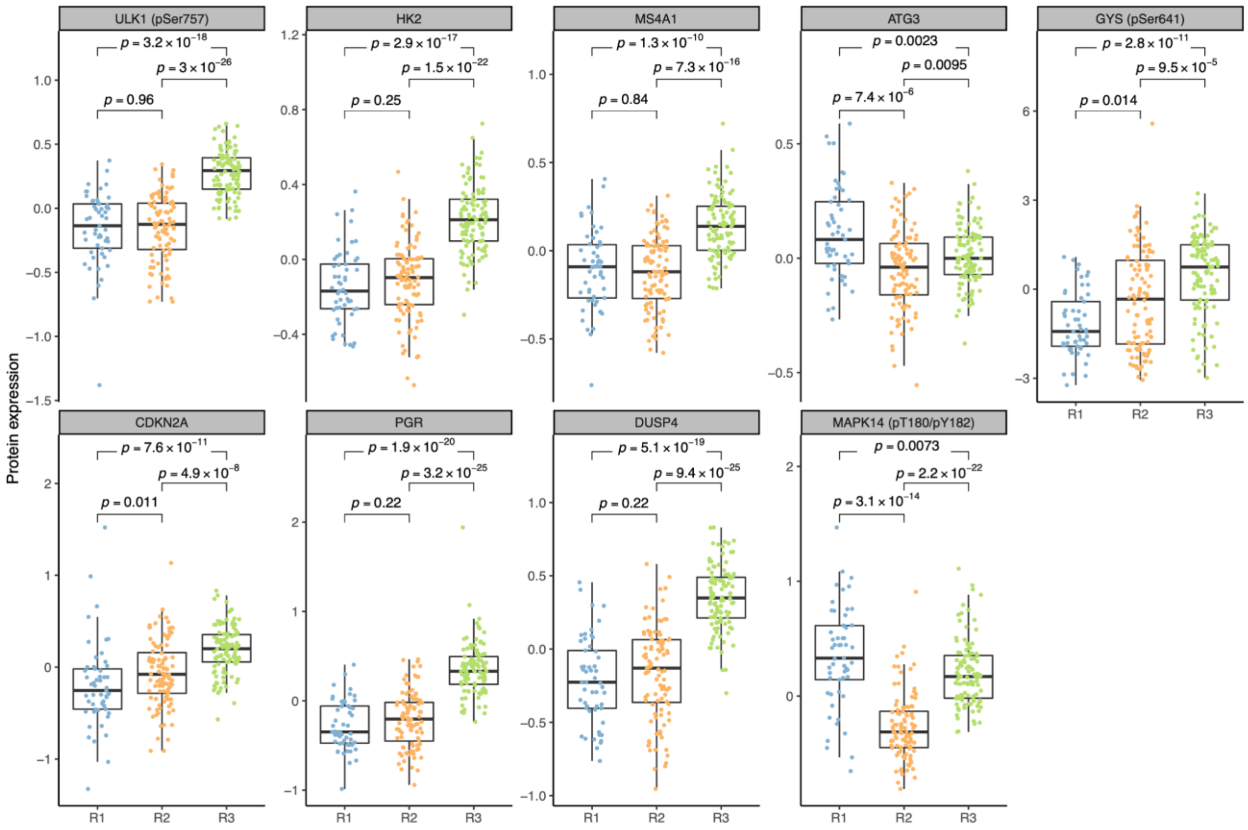

Supplementary Figure 9. Expression levels of good-prognostic proteins. ***, $p < 0.001$; **, $p < 0.01$; *, $p < 0.05$; NS, $p \geq 0.05$. The p-values were computed using two-sided Wilcoxon rank sum test. Sample sizes are n = 53 in R1, n = 100 in R2, and n = 106 in R3. Center of box shows the median. Lower and upper bounds of box are the first and third quartiles, respectively. Minima and maxima are the farthest values within 1.5 times the inter-quartile range from the bounds of box. Whiskers are drawn from the bounds of box to the extrema. Source data are provided as a Source Data file.

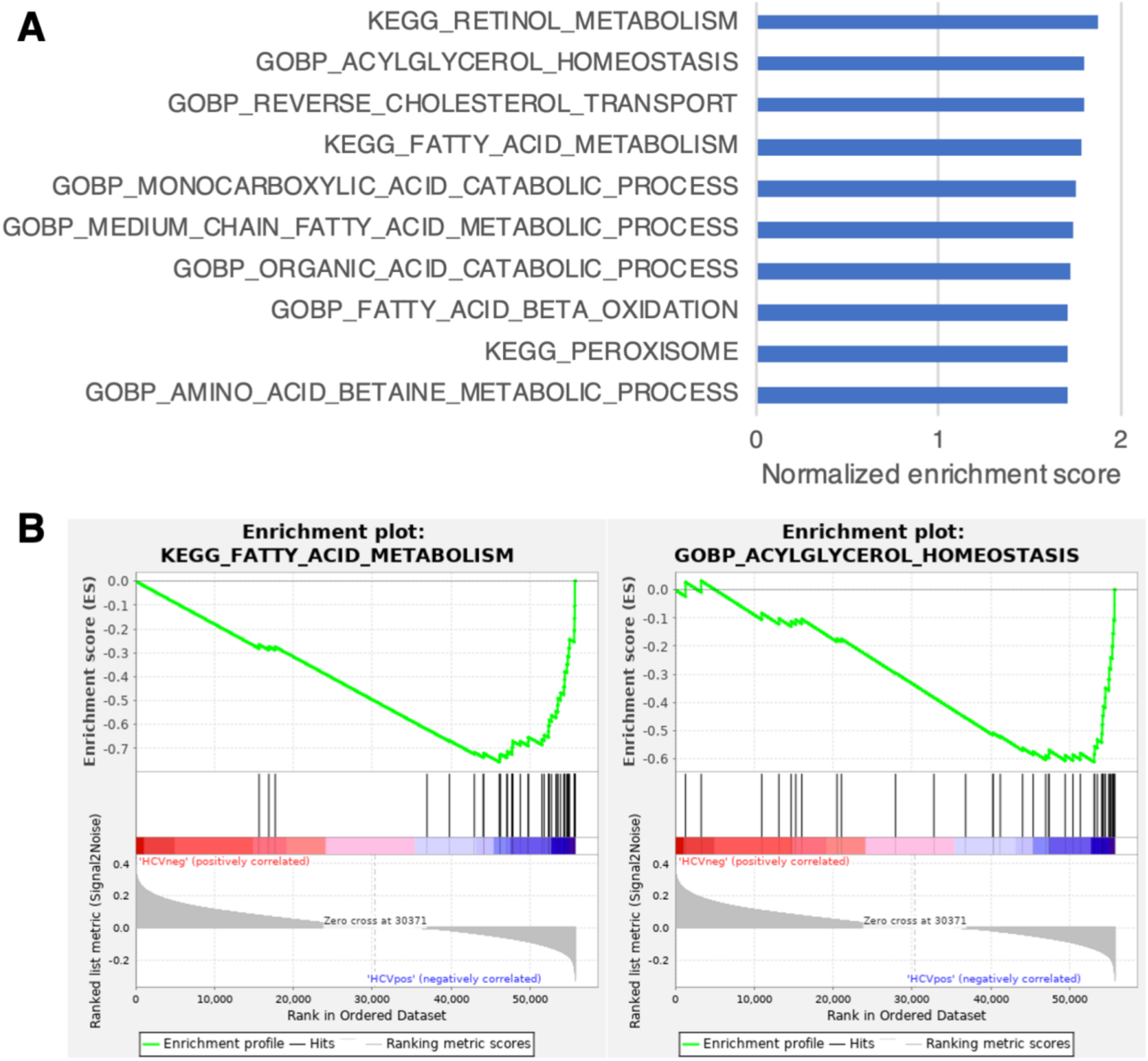

Supplementary Figure 10. Gene set enrichment analysis between HCV-positive and HCV-negative tumors. Enrichment of the GO Biological Process and KEGG pathways in RNA-seq of 234 primary liver cancers were analyzed using the GSEA module (version 20.3.5) of the GenePattern server (https://www.genepattern.org/). (**A**) Top 10 gene sets enriched in HCV-positive tumors. (**B**) Example enrichment plots for lipid metabolism. Source data are provided as a Source Data file.
