# Supplementary material for: Proteo-genomic characterization of virus-associated liver cancers reveals potential subtypes and therapeutic targets
Source: Nat Commun. 2022 Oct 29;13:6481. doi: 10.1038/s41467-022-34249-x (PMC9617926; doi:10.1038/s41467-022-34249-x)
Supplement: Supplementary file 3 — Description of Additional Supplementary Files [file 41467_2022_34249_MOESM3_ESM.pdf]

Description of Supplementary Data Files

Supplementary Data 1. Proteomic classification and clinical information of 259 Japanese patients with liver cancer.

Supplementary Data 2. A list of 293 antibodies that were used for the RPPA measurement of RIKEN dataset

Supplementary Data 3. RPPA data of 259 liver cancers.

Supplementary Data 4. Member proteins of pathways.

Supplementary Data 5. The concentration of pVEGFR2 and pFGFR4 measured by ELISA.
