# Supplementary material for: Proteo-genomic characterization of virus-associated liver cancers reveals potential subtypes and therapeutic targets
Source: Nat Commun. 2022 Oct 29;13:6481. doi: 10.1038/s41467-022-34249-x (PMC9617926; doi:10.1038/s41467-022-34249-x)
Supplement: Supplementary file 9 — Reporting Summary [file 41467_2022_34249_MOESM9_ESM.pdf]

nature portfolio

Corresponding author(s): Hidewaki Nakagawa

Last updated by author(s): Oct 18, 2022

Reporting Summary

Nature Portfolio wishes to improve the reproducibility of the work that we publish. This form provides structure for consistency and transparency in reporting. For further information on Nature Portfolio policies, see our Editorial Policies and the Editorial Policy Checklist.

Statistics

For all statistical analyses, confirm that the following items are present in the figure legend, table legend, main text, or Methods section.

n/a	Confirmed	
☐	☒	The exact sample size (*n*) for each experimental group/condition, given as a discrete number and unit of measurement
☐	☒	A statement on whether measurements were taken from distinct samples or whether the same sample was measured repeatedly
☐	☒	The statistical test(s) used AND whether they are one- or two-sided<br>*Only common tests should be described solely by name; describe more complex techniques in the Methods section.*
☐	☒	A description of all covariates tested
☐	☒	A description of any assumptions or corrections, such as tests of normality and adjustment for multiple comparisons
☐	☒	A full description of the statistical parameters including central tendency (e.g. means) or other basic estimates (e.g. regression coefficient) AND variation (e.g. standard deviation) or associated estimates of uncertainty (e.g. confidence intervals)
☐	☒	For null hypothesis testing, the test statistic (e.g. *F*, *t*, *r*) with confidence intervals, effect sizes, degrees of freedom and *P* value noted<br>*Give P values as exact values whenever suitable.*
☐	☒	For Bayesian analysis, information on the choice of priors and Markov chain Monte Carlo settings
☐	☒	For hierarchical and complex designs, identification of the appropriate level for tests and full reporting of outcomes
☐	☒	Estimates of effect sizes (e.g. Cohen's *d*, Pearson's *r*), indicating how they were calculated

Our web collection on statistics for biologists contains articles on many of the points above.

Software and code

Policy information about availability of computer code

Data collection	The whole genome sequencing and RNA-seq data generated in this study have been deposited in NBDC under the accession numbers JGAD000228 and JGAD000229 (https://humandbs.biosciencedbc.jp/en/hum0158-v2). RPPA data of RIKEN liver cancers are deposited in The Cancer Proteome Atlas Portal (https://tcpaportal.org/tcpa/download.html) with an accession "TCPA00000009".<br>Somatic mutation calls of RIKEN liver cancers are available at https://dcc.icgc.org/releases/PCAWG. A list of pan-cancer driver genes is available at the Synapse website (https://www.synapse.org/#!Synapse:syn11050201). Raw RNA-seq reads of RIKEN liver cancers are available at controlled access database European Genome-phenome Archive (https://ega-archive.org/datasets/EGAD00001001880).<br>RPPA data of The Cancer Genome Atlas Liver Hepatocellular Carcinoma (TCGA-LIHC) is available at The Cancer Proteome Atlas Portal (https://tcpaportal.org/). Archived version of MSigDB v6.2 is available at the MSigDB download site (http://www.gsea-msigdb.org/gsea/downloads_archive.jsp).<br>CCLE data is available from https://depmap.org/portal/download/. CTRP data is available from https://portals.broadinstitute.org/ctrp.v2.1/.
Data analysis	PPA signal intensities were normalized using SuperCurve (v1.5.0). The following publicly available software was used for data analysis: TopHat2 (v2.1.1), HTSeq (v0.6.1), R (v3.6.0), ConsensusClusterPlus (v1.46.0), sva (v3.32.1), caret (v6.0-90), ComplexHeatmap (v2.0.0), ggpubr (v0.2), survival (v2.44-1.1), survminer (v0.4.3), ESTIMATE (v1.0.13), NearestTemplatePrediction (v4), ssGSEAProjection (v9.1.1 beta), ImageJ (v15.3)

For manuscripts utilizing custom algorithms or software that are central to the research but not yet described in published literature, software must be made available to editors and reviewers. We strongly encourage code deposition in a community repository (e.g. GitHub). See the Nature Portfolio guidelines for submitting code & software for further information.

Data

Policy information about availability of data

All manuscripts must include a data availability statement. This statement should provide the following information, where applicable:

- Accession codes, unique identifiers, or web links for publicly available datasets
- A description of any restrictions on data availability
- For clinical datasets or third party data, please ensure that the statement adheres to our policy

The raw whole genome sequencing and RNA-seq data generated in this study have been deposited in NBDC under the accession numbers JGAD000228 and JGAD000229 (https://humandbs.biosciencedbc.jp/en/hum0158-v2). Somatic mutation call data for single nucleotide variants, insertions and deletions, copy number alterations, and structural variants called by the Pan-Cancer Analysis of Whole Genomes (PCAWG) project[14] are available in ICGC DCC (https://dcc.icgc.org/releases). RPPA data of this study is available at The Cancer Proteome Atlas (TCPA) under the accession number TCPA00000009, as well as TCGA liver cancer (LIHC) data set (https://tcpaportal.org/tcpa/). The results of ELISA for pVEGFR2 and pFGFR4 are available in Supplementary Data 5. CCLE data is available from https://depmap.org/portal/download/ [31]. CTRP data is available from https://portals.broadinstitute.org/ctrp.v2.1/ [32]. Source data are provided in this paper as a Source Data file. The remaining data are available within the Article, Supplementary Information or Source Data file.

Human research participants

Policy information about studies involving human research participants and Sex and Gender in Research.

Reporting on sex and gender	We collected gender information for this study and gender is self-reported.
Population characteristics	Japanese population
Recruitment	We recruited liver cancer patients in our collaboration hospitals randomly and once we obtained IC from patients, we stored their samples and analyzed them.
Ethics oversight	This study was approved by IRBs of RIKEN, Hiroshima University Hospital, and Wakayama Medical University Hospital.

Note that full information on the approval of the study protocol must also be provided in the manuscript.

Field-specific reporting

Please select the one below that is the best fit for your research. If you are not sure, read the appropriate sections before making your selection.

☒ Life sciences ☐ Behavioural & social sciences ☐ Ecological, evolutionary & environmental sciences

For a reference copy of the document with all sections, see nature.com/documents/nr-reporting-summary-flat.pdf

Life sciences study design

All studies must disclose on these points even when the disclosure is negative.

Sample size	We previously collected and analyzed 300 samples by genome sequencing (Nature Genet 2016) and this time we selected 240 samples whose frozen tissues were still available among them.
Data exclusions	No data exclusion
Replication	RPPA data of The Cancer Genome Atlas Liver Hepatocellular Carcinoma (TCGA-LIHC) was used as a validation dataset.
Randomization	we selected liver cancer patients randomly.
Blinding	We were blinded to group allocation during our proteomics and genomic analyses.

Reporting for specific materials, systems and methods

We require information from authors about some types of materials, experimental systems and methods used in many studies. Here, indicate whether each material, system or method listed is relevant to your study. If you are not sure if a list item applies to your research, read the appropriate section before selecting a response.

Materials & experimental systems

n/a	Involved in the study
☐	☒ Antibodies
☐	☒ Eukaryotic cell lines
☒	☐ Palaeontology and archaeology
☒	☐ Animals and other organisms
☒	☐ Clinical data
☒	☐ Dual use research of concern

Methods

n/a	Involved in the study
☒	☐ ChIP-seq
☒	☐ Flow cytometry
☒	☐ MRI-based neuroimaging

Antibodies

Antibodies used	For RPPA, a set of 293 validated antibodies were individually applied to the slides (Set 142, the MDACC RPPA Core; Supplementary Data2). The constantly updated list of validated antibodies are available at the RPPA Core Facility, MDACC (https://www.mdanderson.org/research/research-resources/core-facilities/functional-proteomics-rppa-core.html).<br>For ELISA, PathScan phospho-VEGFR2 antibody ELISA kit (Signaling Technology, #7824) and PathScan phospho-FGFR4 ELISA kit (Cell Signaling Technology, #69193) were used.<br>For western blot, we used phospho-p70S6 kinase (Cell Signaling Technology cat#9234, 1:500 dilution), phosphor-Akt (Ser473) (Cell Signaling Technology cat#4058, 1:500 dilution), Phospho-HER2/ErbB2 (Tyr1196) (Cell Signaling Technology cat#6942, 1:500 dilution), Phospho-MEK1/2 (Ser217/221) (Cell Signaling Technology cat#9154, 1:500 dilution), GAPDH (D16H11) (Cell Signaling Technology cat#5174, 1:10000 dilution), and the secondary antibody to Anti-rabbit IgG HRP-linked Antibody (Cell Signaling Technology catalog#7074, 1:2000 dilution).<br>For HC, the tissue sections were stained by DAKO Envision FLEX-LCA (2B11+PD7/26) kit, for CD45, and the tissue sections were incubated with prepared CD4 antibody (Ventana cat# 790-4423, 1:1) or prepared CD8 antibody (Ventana cat# 790-4460, 1:1).
Validation	Validation data of each antibody used for RPPA was described at at the RPPA Core Facility, MDACC (https://www.mdanderson.org/research/research-resources/core-facilities/functional-proteomics-rppa-core.html). Validation of the antibodies used for Western blots and ELISA were described at the website of Cell Signaling Technology (https://www.cellsignal.jp/products/primary-antibodies).

Eukaryotic cell lines

Policy information about cell lines and Sex and Gender in Research

Cell line source(s)	HCC cell lines, HepG2, JHH1, JHH4, JHH5, JHH6 and SUN398, were obtained from JCRB Cell Bank and ATCC.
Authentication	Cell lines were authorized by JCRB Cell Bank and ATCC.
Mycoplasma contamination	We confirmed MP-negative in the cell lines.
Commonly misidentified lines<br>(See ICLAC register)	no misidentified lines
